# The Influence of the Addition of Microsilica and Fly Ash on the Properties of Ultra-High-Performance Concretes

**DOI:** 10.3390/ma18010028

**Published:** 2024-12-25

**Authors:** Anna Szcześniak, Jarosław Siwiński, Adam Stolarski, Artur Piekarczuk, Barbara Nasiłowska

**Affiliations:** 1 Faculty of Civil Engineering and Geodesy, Military University of Technology, 00-908 Warsaw, Poland; jaroslaw.siwinski@wat.edu.pl (J.S.); adam.stolarski@wat.edu.pl (A.S.); 2 Instytut Techniki Budowlanej, 00-611 Warsaw, Poland; a.piekarczuk@itb.pl; 3 Institute of Optoelectronics, Military University of Technology, 00-908 Warsaw, Poland; barbara.nasilowska@wat.edu.pl

**Keywords:** ultra-high-performance concrete (UHPC), microsilica (MS), fly ash (FA), experimental tests, physical properties, mechanical parameters

## Abstract

The paper presents experimental studies on the influence of a simultaneous, appropriately proportioned combination of microsilica and fly ash additives on the physical and mechanical properties of ultra-high-performance concretes (UHPCs). Concrete mixtures with the addition of microsilica in the amount of 6.7–14.7% and fly ash in the amount of 8.3–26.7% were analyzed, assuming a constant content of cement, water and superplasticizer. Experimental studies were carried out regarding the consistency of the fresh concrete mixtures and on the compressive strength, flexural strength, tensile splitting strength, secant modulus of elasticity, depth of penetration of water under pressure into hardened concrete and water absorption. The analysis of mechanical properties was carried out during a long maturation period from 2 to 90 days. Additionally, the influence of the cost of component materials on the final cost of concrete was taken into account. The test results indicate the effectiveness of the use of microsilica and fly ash additives in ultra-high-performance concretes and possible directions for optimizing their proportions in order to achieve the intended physical and mechanical properties. The best strength properties were obtained for concrete containing 16.7% fly ash and 13.3% microsilica. The highest resistance to water penetration and absorption under pressure was characterized by concretes containing an increased content of microsilica up to 14.7%.

## 1. Introduction

Sustainable development in the field of building materials engineering requires maintaining a balance between the rational use of commonly available materials and ensuring their high mechanical properties and durability [1]. The use of concrete with high strength properties and high durability is in line with the trend of sustainable development of building materials, provided that cement consumption is limited and efficient waste or recycled additives are used [2,3,4]. The economic aspect and the related need to rationalize the production costs of high-performance concrete are also important, as they are often a decisive condition for the application of a given solution in practice [5].

Fly ash (FA) is a by-product of hard coal combustion in power plants and combines heat and power plants using blast furnace technologies [6]. This pozzolanic material with spherical shape particles is mainly composed of S_i_O_2_ (<80%), Al_2_O_3_ (<55%) and CaO (<52%) [7]. Fly ash as an additive to concrete increases the workability of fresh concrete mix [7,8], extends the setting time of concrete [9], and reduces the heat of hydration [10] and autogenous shrinkage [11]. Moreover, fly ash increases the tightness of hardened concrete [12], but may delay or reduce the strength of concrete [9,13,14].

Microsilica (MS) in the form of silica fume is a by-product generated during the production of silicon metal in electric arc furnaces [15]. This ultrafine material with spherical particle shapes consists of 85–95% SiO_2_ [16]. Microsilica used as an additive to concrete seals the concrete structure, increases compressive strength and reduces water absorption [17,18,19,20]. In terms of sealing the concrete structure, microsilica is considered one of the most effective materials. Both fly ash and microsilica are commonly used as a partial replacement for cement [21].

The research by Bahedh and Jaafar indicated the advisability of using fly ash as a cement substitute in amounts of up to 40% in high-performance concretes, [8]. In [22], Golewski and Szostak showed that the method of increasing the early strength of concrete containing coal fly ash is the use of nano-admixtures based on C-S-H phase activators.

The authors in [23] confirmed that the use of silica fume in the amount of 15% of the cement mass gives the greatest increase in strength of High-Strength Concrete (HSC). Chan and Chu in [24] indicated that silica fume content in the range of 20–30% of the cement mass is optimal for increasing the bond strength of reactive powder concrete with steel fibers. Chen et al. in [25] found that the optimum silica fume content is 30% for ultra-high-performance reactive powder concrete with 2% steel fiber content. The results of research conducted by Wu et al. [26] showed that in alkali-activated slag–fly ash concrete the highest values of compressive strength, splitting tensile strength and flexural tensile strength were obtained with a microsilica content of 6%.

UHPC is characterized by very high strength properties, ductility, durability and toughness [27]. However, its use requires special conditions for the preparation, production and maturation of concrete, which is also associated with a higher price. Modern solutions of concrete structural systems focus on the use of UHPC as local reinforcement of the most loaded zones [28,29] or in structures exposed to accidental loads [30,31,32].

A very important issue is the rational selection of concrete components due to their cost, which is emphasized by Shah et al. in [5]. According to the data given in [33,34], the price of cement is twice as high as the price of fly ash, while the price of microsilica is almost twenty times higher than the price of fly ash. The addition of steel fibers also causes a significant increase in the price of concrete [33]. In the work [33], economical UHPC mixtures used 5.5% of microsilica additive and 30–40% of fly ash and required thermal curing.

Ultra-high-performance concretes require the use of a significant amount of cement, the production of which is a highly energy-intensive process and therefore has a large carbon footprint. Therefore, in this work, the aim is to obtain a concrete mixture with high strength properties, with limited consumption of cement and expensive microsilica, while maintaining a consistency that allows for easy workability and compaction even in concrete elements with complex reinforcement arrangements. The composition of the concrete mix was developed taking into account the durability of the concrete, including tightness and resistance to water penetration.

Previous studies have focused on analyzing the separate effects of fly ash or microsilica addition as a partial substitute for cement on UHPC properties in different strength ranges. Consequently, the results obtained were the result of both the use of the additive and the limitation of the amount of cement. In this study, an analysis of the influence of the simultaneous combination of fly ash and microsilica additives on the properties of ultra-high-performance concrete was carried out. Assuming the constant content of cement, water and superplasticizer admixtures, the influence of a mutual proportion of the fly ash and microsilica on the physical properties of the fresh concrete mixture (consistency) and on the mechanical properties of hardened concrete (compressive strength, flexural tensile strength, splitting tensile strength, secant modulus of elasticity, depth of penetration of water under pressure and water absorption) was investigated. The research carried out in this study allows for an in-depth analysis of the direct impact of the use of these additives on the physical and mechanical properties of UHPC and the rationalization of their use. Since the available literature does not exhaust the issues of using an additive in the form of a combined mixture of fly ash and microsilica in UHPC, especially taking into account the influence of their proportions, the approach proposed in this paper is a new extension of the research area on UHPC properties. In addition, the conducted cost analysis complements the rationalization of the use of fly ash and microsilica additives in the UHPC mixture. A comparative analysis of mechanical and physical properties was carried out on the basis of experimental tests of UHPC concrete with optimized content of fly ash (16.7% of cement mass) and microsilica (13.3% of cement mass), and three modified concrete recipes. In the first modified recipe, the fly ash content was increased to 26.7%, and the microsilica content was reduced to 6.7%. In the second modified recipe, the microsilica content was increased to 14.7% and the fly ash content was reduced to 8.3%. In the third modified recipe, the addition of fly ash and microsilica was increased to 26.7% and 16.7%, respectively, while the amount of aggregate was reduced.

## 2. Materials

**Figure 1:**
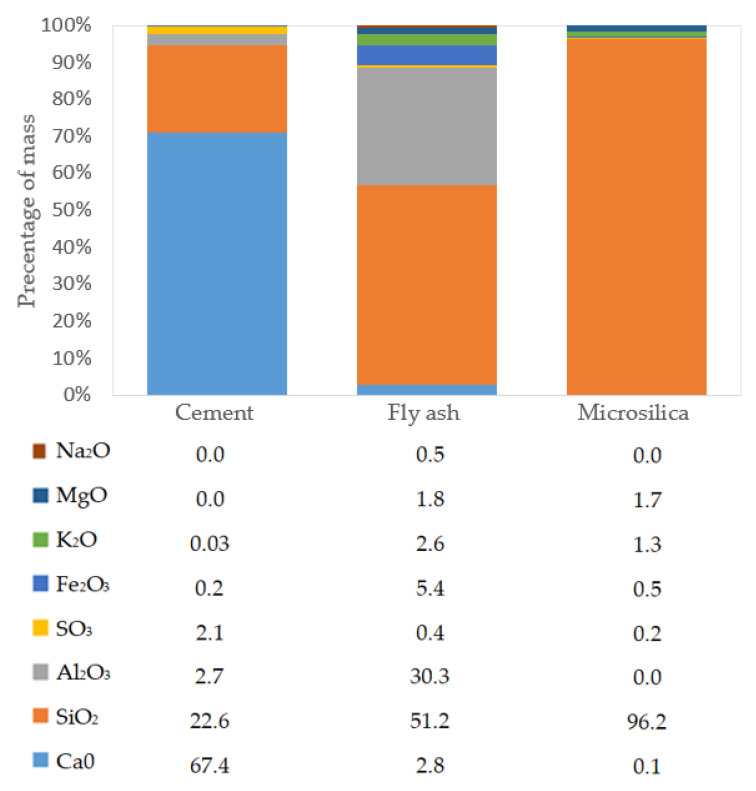
Chemical composition of the cement, fly ash and microsilica.

Experimental studies were carried out for four concrete recipes with constant content of cement, water and superplasticizer. The design assumptions assumed the development of a basic composition of a concrete mix based on basalt aggregate up to 2 mm and sand, with the compressive strength of hardened concrete after 28 days of maturation of at least 120 MPa. In order to enable the practical use of the concrete mixture in construction, especially in structural elements with complex reinforcement systems, a consistency class of at least S3 was adopted, for which the cone slump is not less than 10 cm. In each recipe, 550 kg of Portland cement CEM 52.5 R was used to produce 1 m^3^ of concrete mix, and the value of the w/c coefficient was 0.27. In the CM1 base concrete, the addition of fly ash is 16.7% of the cement mass and the addition of microsilica is 13.3% of the cement mass. Fly ash from hard coal combustion with a fineness below 12% and a loss on ignition below 5% [35] is used in concrete mixtures, which meets the requirements of the EN 450-1 standard [36]. The chemical composition of cement (CEM), fly ash (FA) and microsilica (MS) in the percentage of their own mass is presented in Figure 1 [35,37,38].

To investigate the effect of fly ash and microsilica content on the physical and mechanical properties of concrete, three further mixture recipes were developed: CM2, CM3 and CM4. The composition of these mixtures was developed by modifying the content of microsilica, fly ash and aggregates of the CM1 base mixture. In the CM2 concrete mix, the microsilica content was reduced by half and replaced proportionally with fly ash and aggregate. In the CM3 mixture recipe, the amount of fly ash was reduced by half and replaced proportionally with microsilica and aggregate. The amount of fly ash used in the CM4 concrete mix was the same as in the CM2 mix, and the amount of microsilica was the same as in the CM3 mix, but with a proportionally reduced aggregate content. Changes to the proportions of components were made in relation to the volume fraction of individual components of the concrete mix. In all of the recipes, the same water/cement ratio w/c = 0.27 was maintained, while the water/binder ratio was in the range w/b = [0.20–0.22], depending on the concrete mix. The compositions of concrete mixtures are presented in Table 1.

During the preliminary tests, concrete mixtures were also analyzed in which the amount of microsilica was completely eliminated and attempts were made to replace it with fly ash or aggregate, but these mixes turned out to be insufficiently plastic. The resulting consistency was unacceptable in terms of implementation due to the impossibility of proper compaction.

## 3. Methods

The tests were carried out for concretes of the CM1, CM2, CM3 and CM4 series in accordance with the applicable standards. The tests of fresh concrete consisted of determining the consistency of the mixture using the slump method. Experimental tests were carried out on hardened concrete to determine the compressive strength, flexural tensile strength, splitting tensile strength, secant modulus of elasticity, the depth of penetration of water under pressure and water absorption. In each test, three samples from each concrete series were analyzed. The samples were matured in water at a temperature of 20 °C in accordance with the EN 12390-2 standard [39].

A simplified methodology for estimating the cost of concrete mixtures based on the cost of component materials was proposed.

### 3.1. Concrete Mix Consistency Test

The consistency of all concrete mixtures was determined by the slump test according to EN 12350-2 standard [40]. The fresh concrete mix was placed in a conical mold in three layers and each layer was compacted using a rod. Immediately after removing the mold, the slump of the mixture was measured. The consistency class was determined based on the slump values on a scale from S1 to S5. After removing the mold, a real conical shape of the concrete mix was initially observed in each series of mixtures, and the total slump test time did not exceed 150 s.

### 3.2. Concrete Compressive Strength Test

The uniaxial compressive strength was determined in accordance with EN 12390-3 standard [41] using a TESTING hydraulic press. The maximal compressive force of the press is 5000 kN. The tests were performed at a constant stress rate 0.7 MPa/s. The tests were performed at a constant stress rate of 0.7 MPa/s. The compressive strength was determined on 100 mm cubic samples after 2, 7, 28, 56 and 90 days of concrete maturation and on 150 mm cubic samples after 28 days of maturation.

(1) $$f_{c}=\frac{F_{c}}{A_{c}},\left(MPa\right)$$

where

$F_{c}$—the maximum compressive force (N);

$A_{c}$—the initial cross-sectional area of the sample (mm^2^).

### 3.3. Concrete Flexural Strength Test

The flexural strength of concrete was determined in a three-point bending test in accordance with EN 12390-5 standard [42]. The tests were performed on rectangular samples with dimensions of 40 mm × 40 mm × 160 mm after 2, 7, 28, 56 and 90 days of concrete curing. The maximum force value causing cracking of the samples was determined using a DZ hydraulic press with a maximum force of 100 kN. The tests were performed at a constant force rate of 0.027 kN/s.

The flexural strength of concrete (which basically means the flexural tensile strength of concrete) was determined using the following equation:

(2) $$f_{ct,fl}=\frac{3F_{cr}l}{2d_{1}d_{2}^{2}},(MPa)$$

where

$F_{cr}$—the maximum force (i.e., cracking force) (N);

l—support rollers spacing (mm);

$d_{1}=d_{2}=40$—dimensions of the cross-section sample (mm).

### 3.4. Concrete Tensile Splitting Strength Test

The tensile splitting strength of concrete was determined on 150 mm cubic samples after 28 days of maturation in accordance with EN 12390-6 standard [43]. The tests were carried out using a PW-MEGA automatic hydraulic press with a constant loading stress rate of 0.05 MPa/s.

The tensile splitting strength was determined according to the following formula:

(3) $$f_{ct}=\frac{2F_{s}}{\pi ld},\;(MPa)$$

where

$F_{s}$—the maximum splitting load (N);

l=d=150—the specimen cross-section dimensions (mm).

### 3.5. Concrete Secant Moduli of Elasticity Test

The initial and stabilized secant moduli of elasticity were determined in accordance with EN 12390-13 standard [44] using Method A. The tests were carried out with dimensions of 150 mm × 300 mm cylindrical samples using a PW-MEGA hydraulic press. The measurement base for determining the strains of each sample was three strain gauges with a length of *L*_0_ = 150 mm. Based on the change in the measurement length Δ*L*, the strain *ε* was calculated using the formula:

(4) $$\varepsilon =\frac{∆L}{L_{0}}$$

where

∆L—the measured length change (mm).

**Figure 2:**
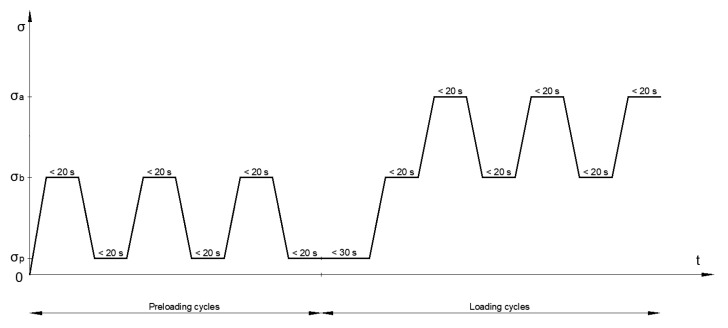
Stress–time cycles for determining initial and stabilized secant modulus of elasticity.

In each sample test, the strain was measured in three preload cycles and three load cycles. In the preloading cycles, the stress range was between the lower stress *σ_p_* = 0.5 MPa and the preload stress *σ_b_* = 0.15 *f_cm_*, and in the loading cycles—between the preload stress *σ_p_* and the upper stress *σ_a_ = f_cm_*/3, where *f_cm_* is the mean value of the compressive strength (Figure 2). In each stress cycle, tests were performed at a constant stress rate of 0.5 MPa/s.

The initial secant modulus of elasticity *E_C_*_,0_ was given by the formula:

(5) $$E_{C,0}=\frac{∆\sigma }{∆\varepsilon_{0}}=\frac{\sigma_{a}-\sigma_{b}}{\varepsilon_{a,1}-\varepsilon_{b,0}}$$

where

$\varepsilon_{a,1}$—the average strain at the upper-stress phase in the first loading cycle;

$\varepsilon_{b,0}$—the average strain at the lower stress phase at the beginning of the loading cycles.

The stabilized secant modulus of elasticity *E_C,S_* is given by the formula:

(6) $$E_{C,S}=\frac{∆\sigma }{∆\varepsilon_{S}}=\frac{\sigma_{a}-\sigma_{b}}{\varepsilon_{a,3}-\varepsilon_{b,2}}$$

where

$\varepsilon_{a,3}$—the average strain at the upper-stress phase in the third loading cycle;

$\varepsilon_{b,2}$—the average strain at the lower stress phase at the beginning of the second loading cycle.

### 3.6. Testing the Depth of Penetration of Water Under Pressure into Hardened Concrete

The water penetration depth under pressure into hardened concrete was tested after 28 days of maturation on 150 mm cubic samples in accordance with EN 12390-8 standard [45]. The tests were carried out under a water pressure of 500 kPa, applied for 72 h, using CONTROLS apparatus. After removal from the apparatus, the samples were split and the maximum depth of water penetration under the tested surface was measured.

Water absorption by the hardened concrete can be also measured by testing the water penetration under pressure into concrete. For this purpose, the weights of the samples immediately before and immediately after exposure to water under pressure were compared, and the difference was expressed as a percentage of the initial weight of the sample.

Water absorption by the hardened concrete is given by the formula:

(7) $$a=\frac{w_{a\;}-w_{i}}{w_{i}}\cdot 100\%$$

where

$w_{i\;}$—initial weight of the sample immediately before;

$w_{a\;}$—weight of the sample immediately after exposure to water under pressure.

### 3.7. Scanning Electron Microscopy Analysis

The observation of the concrete microstructure was performed using a Scanning Electron Microscopy (SEM) Quantana 250 FEG SEM, FEI, Hillsboro, OR, USA, [46]. A backscattered detector (ETD-BSE, FEI, Hillsboro, OR, USA) with an accelerating voltage of 10 kV was used to acquire SEM images. In order to improve the secondary electron emission coefficient as well as the electrical and thermal conductivity of the tested samples, a 6.14 nm layer of gold was sprayed on the surface of the concrete samples before testing. This process was performed using a Leica ACE 600 high-vacuum sputtering machine (Wetzlar, Germany). The analysis was performed for hardened concrete after 28 days of maturation.

### 3.8. Cost Estimation of the Concrete Mix and Its Components

In order to present the degree of profitability of modifying the composition of a concrete mix by changing the content of microsilica and fly ash, an analysis of the costs of the component materials necessary to produce 1 m^3^ of the concrete mix was carried out. It was assumed that the costs of concrete mixtures do not include the price of water due to its equal consumption in each mix. Due to significant differences in the prices of concrete mix components, the analysis of the impact of their cost on the final price of 1 m^3^ of concrete mix was carried out based on the proportion of the prices of component materials to the price of cement.

This relationship is defined by the following dimensionless cost coefficients of the component materials:

(8) $$\upsilon_{k}=\frac{p_{k}}{p_{1}}=\frac{p_{k}}{p_{CEM}}$$

where

$k=\left\{1,K\right\}=\{CEM,FA,MS,B1,B2,S\}$—number of the component material;

K=6—the number of all component materials used in the cost analysis;

$p_{k}$—the price of the component materials.

The mass fraction of the component materials in relation to the mass of cement in 1 m^3^ of a given concrete mix is determined using the following dimensionless mass coefficients of the component materials:

(9) $$\mu_{CMj,k}=\frac{m_{k}}{m_{1}}=\frac{m_{k}}{m_{CEM}}$$

where

$j=\left\{1,J\right\}=\{CM1,CM2,CM3,CM4\}$—number of concrete mix CM*j*;

J=4—number of all analyzed concrete mixtures;

$m_{k}$—mass of component materials in 1 m^3^ of concrete mix, according to Table 1.

Based on the mass coefficient of the component material (Equation (9)) and the cost coefficient of the component materials (Equation (8)), the component cost coefficient in a given concrete mixture CM*j* is determined by the formula:

(10) $$V_{CMj,k}=\mu_{j,k}\upsilon_{k}$$

In turn, the mixture cost factor is determined based on the relationship:

(11) $$W_{CMj}=\sum_{k=1}^{K}V_{CMj,k}$$

Finally, the cost factors of the CM2, CM3 and CM4 mixtures were compared with the cost factor of the CM1 base mixture, and the result was presented in the form of a global cost indicator that allows conclusions to be drawn on the price relationships of the individual concrete mixtures:

(12) $$\psi_{CMj}=\frac{W_{CMj}}{W_{CM1}}$$

## 4. Results and Discussion

### 4.1. Consistency

The results of the tests of the consistency of concrete mixtures and the determination of their class are presented in Table 2.

The base concrete mixture is characterized by a high degree of fluidity, allowing it to be classified as an S4 consistency class, and the cone fall is 210 mm. Reducing the amount of microsilica and replacing it with fly ash in the CM2 mixture resulted in a significant reduction in the fluidity degree of the concrete mixture. The cone slump of the CM2 mixture is 90 mm, which corresponds to the consistency class S2. Reducing the amount of fly ash and replacing it with microsilica also resulted in a decrease in the fluidity degree of the CM3 concrete mixture, but to a much lesser extent than that observed in the CM2 mixture. The cone slump of the CM3 concrete mix is lower than that of the CM1 base mix and is 175 mm, but the consistency class remains unchanged, i.e., S4. Importantly, the use of an increased amount of microsilica and fly ash with a reduced amount of aggregate in the CM4 concrete mix allowed for maintaining the same consistency class as in the CM1 concrete mix.

### 4.2. Compressive Strength

**Figure 3:**
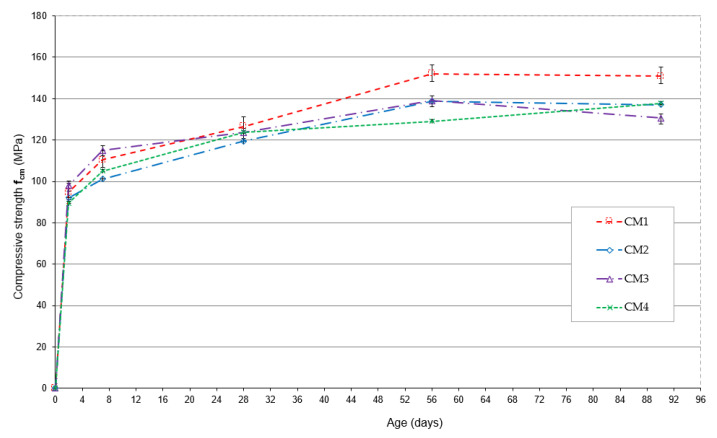
Compressive strength of concrete as a function of maturation time.

In the early maturation period, both after 2 and 7 days, the highest compressive strength is observed in the CM1 base concrete, Figure 3.

After two days of maturation, the compressive strength of CM3 concrete is only 1% lower than that of CM1 concrete, while that of CM2 and CM4 concrete is 4% and 5% lower, respectively. After 7 days of maturation, there is a noticeable delay in achieving the compressive strength of CM2 concrete, which is 14% lower than that of CM1 concrete. The compressive strength of CM3 and CM4 concretes is similar, and their values are lower than those of CM1 concrete by 4% and 6%, respectively. After 28 days of maturation, the highest compressive strength is observed in CM4 concrete, which contained the largest amount of microsilica and fly ash, with a reduced amount of aggregate. The compressive strength of CM1, CM2 and CM3 concrete is lower by 3%, 4% and 2%, respectively, compared to the compressive strength of CM4 concrete.

**Figure 4:**
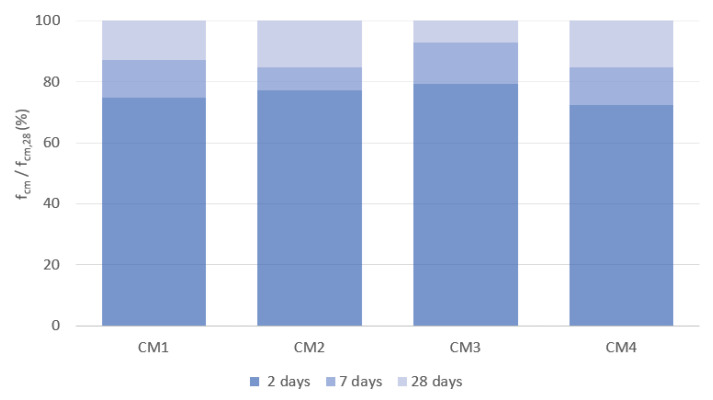
Changes in early compressive strength of concretes.

Figure 4 shows the increase in compressive strength over time, expressed as a percentage of the compressive strength obtained on day 28 of maturation.

In the early maturation period, the greatest increase in compressive strength is observed for CM1 concrete, which, after 2 days, reaches 78% of the compressive strength obtained after 28 days, and after 7 days of maturation, this strength is already 91% of the 28-day strength. After two days of maturation, CM2 and CM3 concretes achieve a compressive strength of 76% of the strength achieved after 28 days of maturation, while after 7 days of maturation, the strength of these concretes is 81% and 87% of the 28-day strength, respectively. CM4 concrete is characterized by the smallest increase in compressive strength after 2 and 7 days, which is 72% and 84% of the compressive strength obtained after 28 days of maturation, respectively. After 56 days of maturation, a significant increase in the compressive strength of CM1, CM2 and CM3 concretes is observed, which, in comparison to their strength after 28 days of maturation, is 20%, 16% and 12%, respectively. The smallest increase of 4% during this period of concrete maturation is observed for CM4 concrete. After 56 days of maturation, the mean compressive strength of the CM1 base concrete is the highest and is 152 MPa. The compressive strength of CM2 and CM3 concretes is almost the same and is 9% lower than the strength of CM1 concrete. The lowest compressive strength after 56 days of maturation is achieved by CM4 concrete, whose strength is 15% lower than the strength of the base CM1 concrete. After 90 days of maturation, the compressive strength of CM1 and CM2 concretes remains almost unchanged, while an increase in the strength of CM4 concrete is observed, the mean value of which is now 137.8 MPa. Interestingly, in the case of CM3 concrete, after 90 days of maturation, a 6% decrease in compressive strength is observed compared to that obtained after 56 days of maturation.

**Figure 5:**
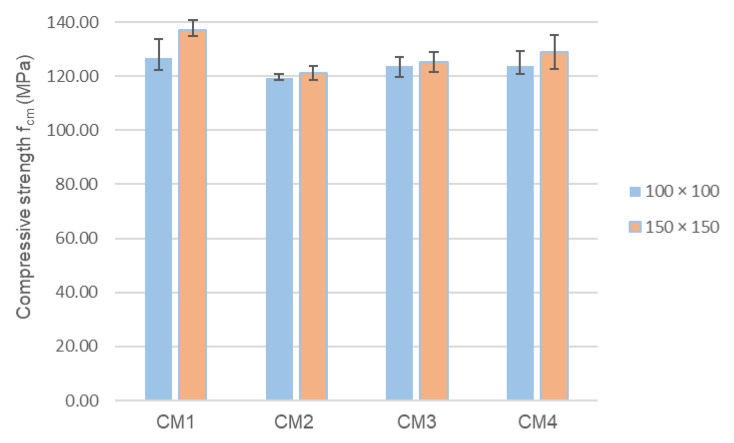
Compressive strength of concrete was determined on cubic samples with an edge length of 100 mm and 150 mm after 28 days of maturation.

Figure 5 shows the results of concrete compressive strength tests determined on cubic samples with an edge length of 150 mm after 28 days of maturation, which were compared with the results obtained for cubic samples with an edge length of 100 mm. For cubic samples with an edge length of 150 mm, the highest mean compressive strength of 136.92 MPa was obtained for CM1 concrete. For these samples, the compressive strength of CM4 concrete is 6% lower, and the strength of CM2 and CM3 concrete is 8.5% and 11% lower, respectively. Interestingly, in the case of all four analyzed concrete series, the compressive strength determined on cubic samples with an edge length of 150 mm is higher than the strength determined on cubic samples with an edge length of 100 mm. In the case of concretes from the CM1 and CM4 series and both the CM2 and CM3 series, these differences are 8%, 4% and only 1%, respectively. This is a surprising phenomenon, especially since, due to the scale effect, the opposite trend could be expected.

The influence of scale effect on compressive strength of UHPC containing steel fibers was presented by Abbas et al., [47]. The compressive strength of concrete determined on 50 mm cubic samples was 9% higher compared to the strength determined on 100 mm cubic samples. However, Tallat [48] notes that, in addition to the size and shape of samples, the third main factor influencing the compressive strength results is the friction at the loaded surfaces of the samples. A comparison of the results for 100 mm, 150 mm, and 200 mm cubic samples, shows an increase in compressive strength with an increase in the lateral dimension of the sample.

The experimental results of Yazici et al. presented in [49] confirmed the validity of using a combination of fly ash and microsilica in reactive powder concretes containing 2% steel fibers. A delay in the compressive strength of concrete after the use of fly ash is observed only in the early curing period after 2 days, while after 28 days, the concrete containing 30% microsilica and 25% fly ash has a strength that is 4% higher compared to concrete without the addition of fly ash.

The results of Wu et al. [26] show that in slag–fly ash concrete containing basalt fibers, the simultaneous increase in silica fume content and a proportionally decreasing fly ash content results in increased strength properties, but only when the silica fume content does not exceed 6%. Increasing the silica fume content to 9% with a proportional reduction in the fly ash content resulted in a decrease in compressive strength, tensile splitting strength and flexural strength. Importantly, in this concrete, each of the strengths was lower than that obtained for concrete without the addition of silica fume. Alsalman et al. [33] showed that in heat-cured UHPC containing 20% silica fume, the compressive strength decreased by 19% after adding 20% fly ash. In UHPC containing 5.5% microsilica, the addition of 30% fly ash caused a 9% decrease in compressive strength. Interestingly, further increasing the fly ash content in the mixture to 40% did not result in deterioration of the composite compressive strength. The compressive strengths of UHPC containing 30% and 40% fly ash were almost identical, and the difference between them did not exceed 1%. However, increasing the fly ash content to 50% resulted in a 43% decrease in the composite compressive strength compared to the composite without fly ash. It should be noted that the tests described in [33] were carried out using special composite curing conditions.

### 4.3. Flexural Strength

**Figure 6:**
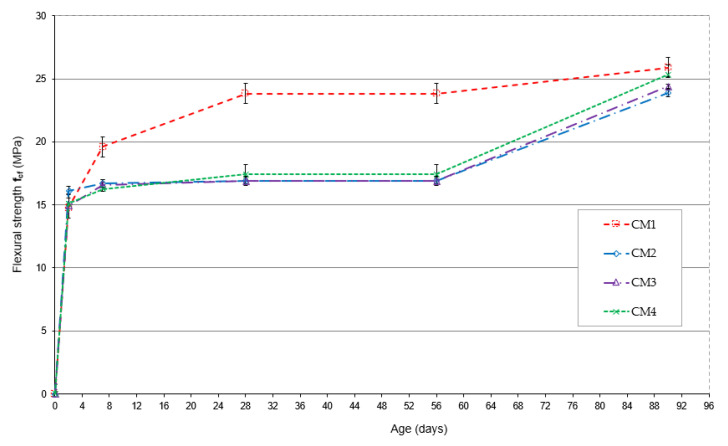
Flexural strength of concretes as a function of maturation time.

The change in flexural strength during the maturation period from 2 to 90 days is shown in Figure 6.

The highest flexural strength after 2 days of maturation is characteristic of CM2 concrete with increased fly ash content, and this strength is 96% of the strength obtained after 28 days of maturation. The flexural strength of CM1, CM3 and CM4 concretes has similar values, but compared to the strength of CM2 concrete, they are 9%, 8% and 7% lower, respectively. After 7, 28, 56 and 90 days of maturation, the CM1 base concrete has the highest flexural strength. The flexural strength of CM1 concrete, compared to the strength of CM2, CM3 and CM4 concretes after 7 days of maturation, is higher by 17%, 16% and 20%, respectively, and after 28 days, by as much as 41%, 44% and 37%. After 56 days of maturation, CM1 concrete achieved a flexural strength higher than CM2 concrete by 21%, CM3 by 26%, and CM4 by 5%. After 90 days of maturation, the difference in the flexural strength of CM1 concrete compared to CM2, CM3 and CM4 concrete decreased to 8%, 6% and 2%, respectively.

CM2 concrete is characterized by the greatest increase in flexural strength during the maturation period. After 2 days of maturation, its flexural strength was 96% of the strength achieved after 28 days of maturation, and 99% after 7 days. The smallest increase in flexural strength during maturation was observed in CM1 concrete, whose strength after 2 days of maturation was 62% of the strength obtained after 28 days of maturation, and 82% after 7 days. The remaining CM3 and CM4 concrete after 2 days of maturation achieved flexural strengths above 86% of the strength after 28 days of maturation and above 93% after 7 days of maturation.

Analysis of the flexural strength test results indicates that the highest strength is characteristic of the CM1 base concrete, whose strength is over 17% higher after 7 days and over 37% higher after 28 days of maturation compared to the strength of the other concretes. The flexural strength of CM2 and CM3 concrete remains at a very similar level after both 7 and 28 days of maturation. The flexural strength of CM4 concrete after 28 days is only 3% higher than that of CM2 and CM3 concrete, whose average strength is identical. CM1 base concrete is also characterized by the greatest delay in strength growth in the earliest 2- and 7-day maturation period. Noteworthy is the CM2 concrete, which, already on the 2nd day of maturation, achieved a flexural strength of 96% of the strength obtained after 28 days. In this concrete, the amount of microsilica was reduced, while the fly ash content was increased to 26.7% of the cement mass. In CM4 concrete containing both increased amounts of fly ash and microsilica, the flexural strength obtained after 2 days was 87% of the strength obtained after 28 days of maturation.

The phenomenon of increasing tensile strength after using a combination of fly ash and microsilica as additives to concrete in comparison to the strength of concrete containing only microsilica was also observed in [49]. In the results of Wu et al. [50], a delay in the flexural strength of UHPC was observed during a long maturation period of up to 90 days, accompanying the increase in fly ash content. The analysis was carried out for a base concrete containing 25% silica fume and 2% steel fibers, which was modified by changing the fly ash content from 20% to 60% as a partial substitute for cement. With the increase in fly ash content, the early flexural strength decreases after 3 and 7 days of maturation, whereas after 28 days and 90 days, an increase in flexural strength is noticeable in UHPC containing fly ash addition compared to the base concrete. The highest flexural strength after 28 days of maturation was obtained for concrete containing 20% fly ash addition and it is 25% higher than the flexural strength of concrete without fly ash, assuming standard maturation conditions.

### 4.4. Tensile Splitting Strength

**Figure 7:**
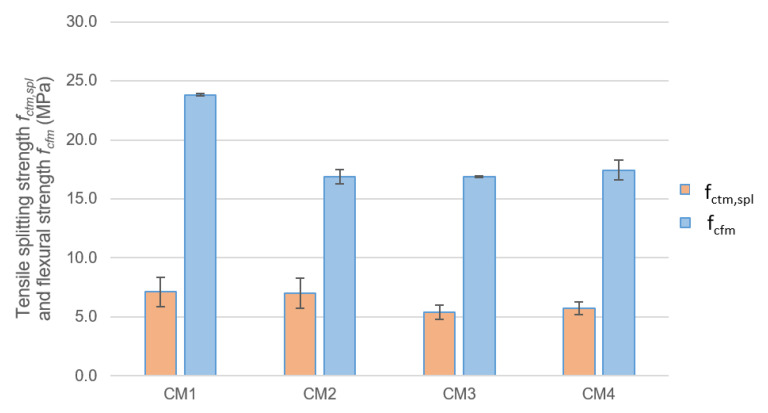
Tensile splitting strength and flexural strength in 28 days of maturation.

The results of the tensile splitting strength tests compared to the flexural strength of concrete after 28 days of maturation are shown in Figure 7. CM1 concrete has the highest splitting tensile strength. The strength of CM2 concrete is only 2% lower. However, the strengths of CM3 and CM4 concrete are 24% and 19% lower, respectively, compared to the CM1 base concrete.

The relationship between the tensile splitting strength and the flexural strength for CM1, CM3 and CM4 concretes are similar and range from 0.3 to 0.33, while for CM2 concrete, this relationship is 0.41. Partial replacement of microsilica with fly ash in CM2 concrete can be considered the most effective modification of the CM1 base concrete in terms of tensile splitting strength.

The results of Yigiter et al. in [51] indicate that in low cement reactive powder concrete (LCRPC) based on bauxite aggregate with constant content of silica fume and steel fibers, the tensile splitting strength decreases with increasing fly ash content. However, the flexural strength increases by 3% compared to the base concrete if the fly ash content is 20%. In turn, the addition of fly ash in the amount of 40% or 60% causes a decrease in flexural strength by 3% and 13%, respectively.

### 4.5. Secant Modulus of Elasticity in Compression

**Figure 8:**
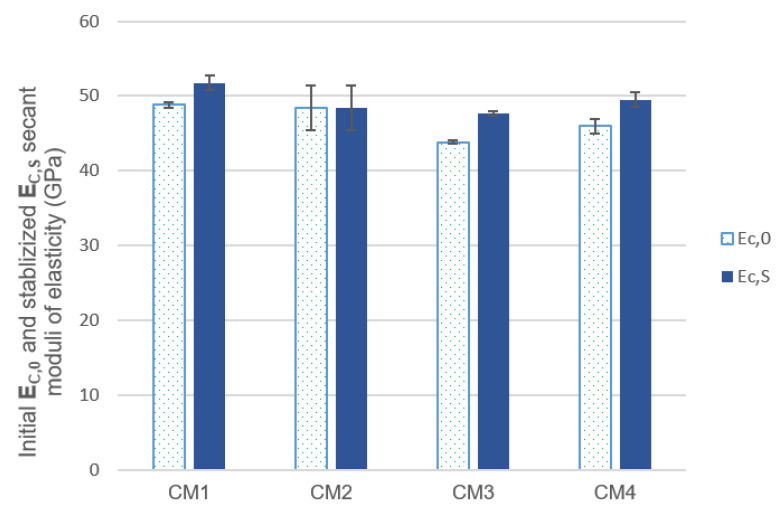
Initial and stabilized secant moduli of elasticity after 28 days of maturation.

The results of the initial and stabilized secant modulus of elasticity in the compression tests are shown in Figure 8. CM1 base concrete has the highest values of these modules. The mean value of the initial secant modulus of elasticity of concrete CM2 is 1% lower than the initial value of the modulus of concrete CM1, while the moduli of concrete CM4 and CM3 are 6% and 10% lower, respectively. The mean value of the stabilized secant modulus of elasticity of concrete CM1 is 6% higher than the mean value of the modulus of concrete CM2 and by 8% and 4%, respectively, than the mean value of the moduli of concrete CM3 and CM4. CM3 concrete is characterized by the lowest mean values of both the initial and stabilized moduli.

The test results indicate that increasing the microsilica content in the CM3 concrete mixture resulted in a greater reduction in the mean value of secant moduli of elasticity compared to the CM2 concrete in which a proportional amount of fly ash was used. This phenomenon is particularly visible when comparing the values of the initial modules of CM2 and CM3 concretes, for which the difference is over 9%, while the difference in the mean values of the stabilized modules does not exceed 2%. Interestingly, the simultaneous use of increased content of microsilica and fly ash in CM4 concrete resulted in an increase in the mean value of the stabilized secant modulus of elasticity by 2% compared to the value of the modulus of CM2 concrete and by 4% compared to the value of the modulus of CM3 concrete. The mean value of the initial modulus of elasticity of CM4 concrete is 5% lower than the mean value of the modulus of CM2 concrete, and compared to CM3 concrete, it is 5% higher.

### 4.6. Depth of Penetration of Water Under Pressure into Hardened Concrete

**Figure 9:**
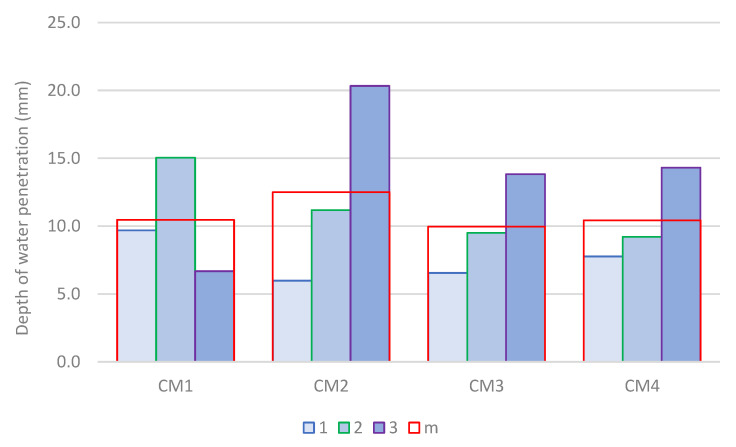
Depth of water penetration under pressure in hardened concrete in 28 days of maturation.

The depth of penetration of water under pressure into hardened concrete after 28 days of maturation is shown in Figure 9. The graph refers to the greatest observed depth of water penetration into the sample, which is marked sequentially with numbers from 1 to 3, and m is the mean value determined from measurements for three samples from each series. In none of the analyzed concrete series was there a penetration depth of water under pressure exceeding 21 mm. Against the background of the research results in the field of integral waterproof concrete [52,53], all analyzed concretes can be recognized as resistant to the penetration of water under pressure. The greatest water penetration depth was determined for sample no. 2 of the CM2 series concrete. The average water penetration depth for this series was also the largest among all analyzed concrete series. CM3 concrete has the smallest average depth of penetration of water under pressure, for which it is 10 mm. In both CM1 and CM4 concrete, this value is 5% higher, while in CM2 concrete, it is 25% higher compared to CM3 concrete.

In each of the analyzed concrete series, the penetration depth of water under pressure did not exceed 21 mm. In light of the research results in the field of integrally waterproof concrete [52,53], all the analyzed concretes can be considered resistant to the penetration of water under pressure. The greatest water penetration depth was determined for sample No. 2 of CM2 concrete. The mean value of water penetration depth for this concrete was also the highest among all analyzed concrete series. CM3 concrete has the lowest mean value of depth of penetration of water under pressure, which is 10 mm. In both CM1 and CM4 concrete, the mean value of depth of penetration of water under pressure is 5% greater, while in CM2 concrete, it is even 25% greater compared to CM3 concrete.

**Figure 10:**
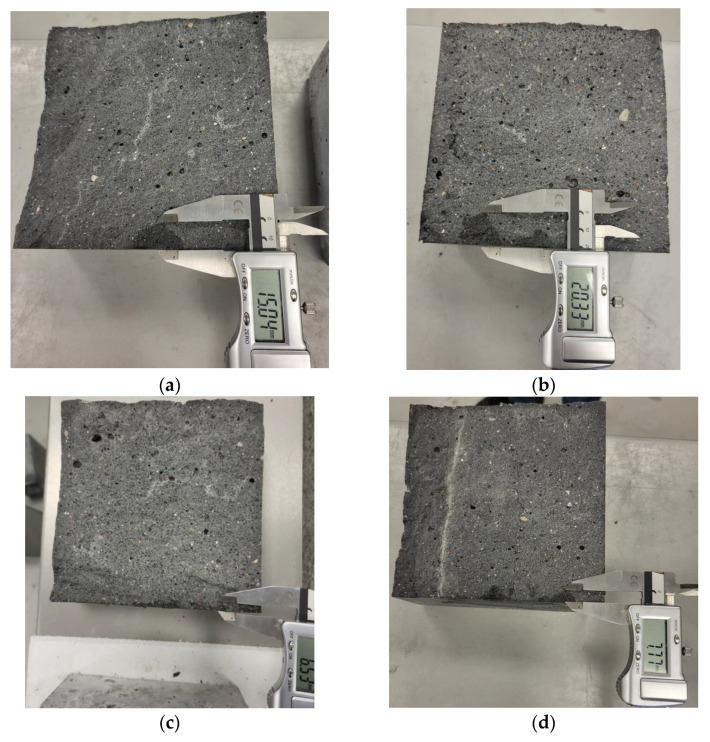
Depth of water penetration under pressure in concrete series: (**a**) CM1, (**b**) CM2, (**c**) CM3, (**d**) CM4.

Analysis of concrete samples subjected to water penetration under pressure showed that in some samples the range of water penetration was local, while in others it was more uniform and had a larger range, Figure 10.

**Figure 11:**
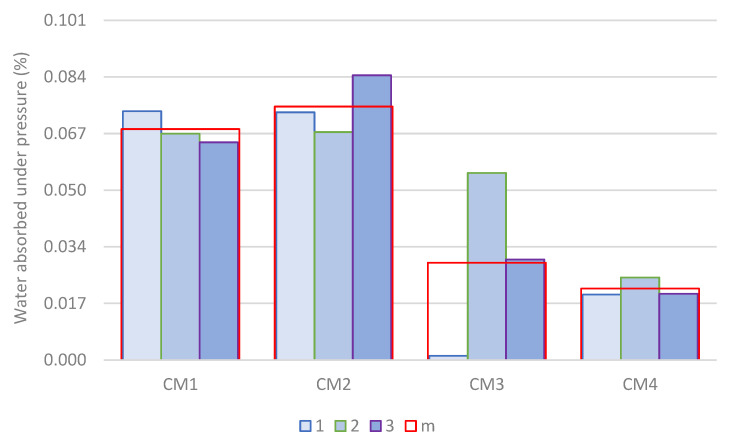
Water absorption under pressure in hardened concrete after 28 days of maturation.

To better describe this phenomenon, the amount of water absorbed by the hardened concrete was measured during a test of water penetration under pressure into concrete. Measurement results according to Equation (7) are shown in Figure 11. Sample numbers and mean values are marked similarly to Figure 9. In the CM1 concrete series, the highest water absorption was observed in sample No. 1. However, in this sample, the penetration depth of water under pressure was 36% less than in sample No. 2, in which the penetration depth was the greatest in the CM1 concrete series. In the CM2 concrete series, sample No. 3 was characterized by both the highest water absorption and the greatest depth of water penetration under pressure. However, sample No. 1, in which the smallest depth of water penetration under pressure was observed, is characterized by greater water absorption than sample No. 2. In the CM3 concrete series, the highest water absorption was observed in sample No. 2, while the greatest depth of water penetration under pressure was observed in sample No. 3. A similar phenomenon was observed in samples No. 2 and No. 3 of the CM4 concrete series.

Comparing the results obtained for each series of concrete, one can observe a clear advantage in limiting water absorption of concretes containing an increased amount of microsilica. The average grain size of microsilica is 100 times smaller than the average grain size of cement. Therefore, microsilica grains can fill the empty spaces between cement grains and fly ash, leading to reduced pores, sealing and increased the density of the concrete matrix. Moreover, the strong pozzolanic properties of microsilica cause the reactive silicon particles to react with calcium hydroxide Ca(OH)_2_, additionally creating a sparingly soluble structure filling the pores [54]. This phenomenon is particularly visible in the CM4 series concrete, in which an additional amount of fly ash was used, but also in the CM3 concrete. CM2 concrete with increased fly ash content and reduced microsilica content is characterized by the lowest resistance to the penetration of water under pressure. These results clearly indicate the sealing properties of microsilica, which increase with increasing microsilica content in the concrete. However, increasing the amount of fly ash to 26.7% of the cement mass, combined with increasing the amount of microsilica to 14.7% of the cement mass, leads to an additional reduction in water absorption by concrete.

The test results presented in [35] indicate a close relationship between the resistance of concrete to the penetration of water under pressure and the resistance of concrete to chemical aggression. Namely, these results clearly indicate that limiting the penetration of water also leads to limiting the penetration of chloride ions into concrete. The research by Wu et al. [26] indicates that the use of silica fume as a partial replacement for fly ash, but only in the range of up to 6%, leads to the sealing of alkali-activated concrete against chloride penetration. Increasing the amount of silica fume to 9% resulted in a deterioration of the concrete’s resistance to chloride ion penetration. In the experimental analysis of concrete resistance to water penetration conducted by Liu et al. in [55], with the increase in fly ash addition as a partial cement substitute, the water penetration depth increases, while capillary water absorption decreases. It should be emphasized, however, that these tests were carried out for concretes based on sea sand and using seawater.

### 4.7. Microstructural Analysis

**Figure 12:**
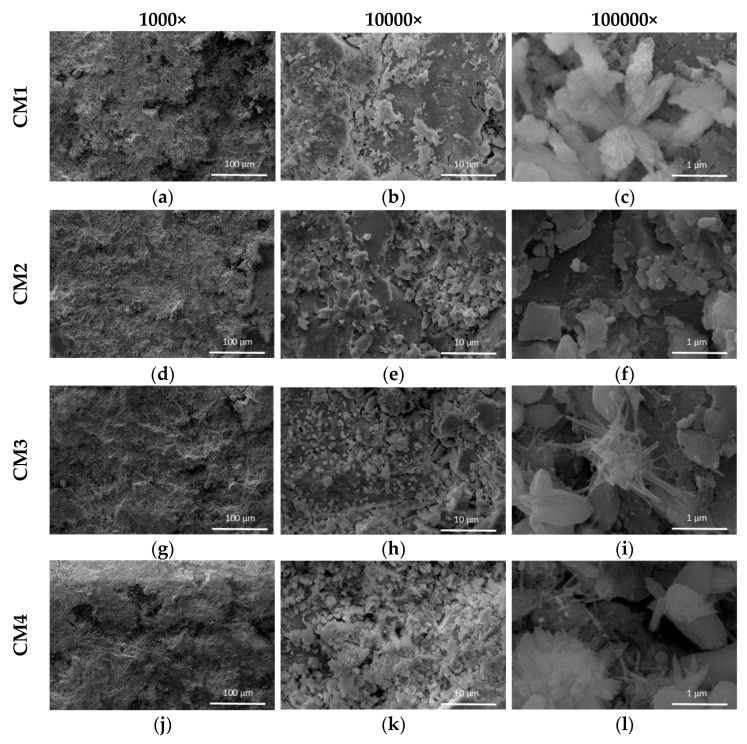
SEM microstructure images of concrete at different magnifications: (**a**) CM1 1000×, (**b**) CM1 10000×, (**c**) CM1 100000×, (**d**) CM2 1000×, (**e**) CM2 10000×, (**f**) CM2 100000×, (**g**) CM3 1000×, (**h**) CM3 10000×, (**i**) CM3 100000×, (**j**) CM4 1000×, (**k**) CM4 10000×, (**l**) CM4 100000×.

The results of the microstructure analysis of concretes carried out using SEM are presented in Figure 12. Imaging was performed for three levels of magnifications, i.e., 1000×, 10000× and 100000×. All concrete samples are characterized by a compact structure. However, in the case of concretes with increased content of microsilica, the structure is more homogeneous. The hydrated cement paste particles C-S-H observed in CM1 concrete form the most compact structure, see Figure 12. In CM3 concrete, hydration products are observed in the form of clusters of needle-shaped crystals, see Figure 12h,i. Crystal structures in this form are characteristic of UHPC concretes and cause densification of the C-S-H gel structure [56]. Needle-shaped crystal structures are also observed in CM4 concrete, but they are more dispersed, see Figure 12k,l. With the increase in microsilica content, an increase in the hydration products in the microstructure of UHPC is observed, which is consistent with other research results [57]. In the case of CM2 concrete, the hydration products have an amorphous structure (Figure 12e,f), which is characteristic of concretes containing a significant addition of fly ash [58]. Importantly, no unhydrated cement grains were found in the analyzed samples, while they are often found in UHPC due to the high cement content [59].

### 4.8. Cost Analysis of the Concrete Mix and Its Components

The results of the cost analysis are presented in Table 3. The basis of the analysis is the cost coefficients of the component materials $\upsilon_{k}$, see Equation (7), determined based on market prices of building materials in Poland in 2024, and the amounts of components of individual concrete mixtures according to Table 1, expressed by $\mu_{CMj,k}$ mass coefficients, see Equation (9). The adopted price proportions of the component materials correspond to the data contained in [33,34]. The cost of concrete mixtures does not include the price of water due to its equal consumption in each mixture and its negligible price.

Analysis of the results shows that the CM2 concrete mix has the lowest cost indicator. The cost indicator of the CM1 concrete mix is more than 14% higher compared to the cost indicator of the CM2 concrete mix. The highest cost indicator is characterized by the CM4 concrete mix containing an increased amount of microsilica and fly ash, which is 17% higher compared to the CM2 concrete mix containing a reduced amount of microsilica and with an increased share of fly ash. The cost indicator of CM3 concrete mix containing an increased addition of microsilica is only 1% lower than the cost indicator of CM4 concrete mix.

Balancing the strength requirements with the production costs of UHPC is an important factor. Alsalman et al. in [33] drew attention to the huge differences in UHPC costs. The production cost of 1 m^3^ of some UHPCs can be almost USD 3000 while the cost of others is almost ten times lower. The main factor determining the high cost of UHPC is the addition of steel fibers and a high silica fume content. Reducing the use of microsilica and increasing the fly ash content have been indicated as directions for reducing UHPC costs. Also, Arman et al. in [27] indicate that one of the main challenges for the effective use of UHPC is to reduce its production costs. As one of the solutions, the authors indicate the use of regional raw materials and waste products in order to reduce the consumption of cement, steel fibers and chemical admixtures.

## 5. Conclusions

The obtained results indicate that microsilica improves the workability of the concrete mixture much more effectively than fly ash. The introduction of microsilica additives with grains many times smaller than fly ash grains results in better fluidification of the concrete mix. The dispersion properties of microsilica cause it to fill the intergranular spaces, creating a specific coating around cement particles. The use of an increased amount of fly ash with a reduced amount of microsilica resulted in a significant reduction in the fluidity of the CM2 concrete mix, and the cone slump decreased by 120 mm compared to the CM1 base mix. Increasing the amount of microsilica with a reduced amount of fly ash also resulted in a decrease in the fluidity of the CM3 concrete mix, but to a lesser extent than in the CM3 mix, and the cone slump decreased by 35 mm compared to the CM1 base mix. The condition for maintaining the highest possible degree of fluidity of the concrete mix was the use of fly ash and microsilica with a mass content in the range MS/FA = 0.55–0.8, which was observed in the consistency test of CM1 and CM4 mixes.

The high pozzolanic activity of microsilica is due to the high content of reactive silicon particles that react with calcium hydroxide CaOH_2_, and the large specific surface area of the pozzolan. These properties lead to an increase in both the early strength and the strength of concrete conditioned for 28 days. The pozzolanic activity of fly ash is much lower, so these processes are much slower. Therefore, when fly ash is added to concrete, a slow increase in strength is observed. Partial replacement of microsilica with fly ash resulted in a slower pozzolanic reaction in the initial maturation period; therefore, a decrease in the compressive strength of CM2 concrete is observed after 2, 7 and 28 days of maturation. Reducing the microsilica content in the CM2 concrete mix from 13.3% of the cement mass to 6.7% while increasing the fly ash content from 16.7% to 26.7% of the cement mass resulted in a 6% decrease in the compressive strength of the concrete after 28 days of maturation.

The pozzolanic properties of microsilica lead to the increased strength of concrete provided that it is used in the appropriate proportion to the remaining components of the concrete mix. The course of the pozzolanic reaction depends on the presence of Ca(OH)_2_, which is formed in the cement hydration process. Therefore, the microsilica content should be adjusted to the cement content. This also applies to other components reacting with Ca(OH)_2_, including fly ash. Excessive microsilica content can lead to a decrease in the strength of concrete, as is observed in the case of the CM3 and CM4 concretes. The research shows that the most effective amount of microsilica is in the range of 6.7–13.3% of the cement mass. Reducing the amount of fly ash from 16.7% to 8.3% of the cement mass while increasing the amount of microsilica from 13.3% to 14.7% of the cement mass resulted in a 2% decrease in the compressive strength of CM3 concrete after 28 days of maturation. The same effect was obtained by simultaneously increasing the amount of microsilica to 14.7% and fly ash to 26.7% of the cement mass in the CM4 mixture.

The use of an increased amount of both microsilica and fly ash causes a significant delay in achieving the flexural tensile strength of concrete both in the early maturation period up to 28 days and after 56 days. In the long-term maturation period, after 90 days, this phenomenon disappears. After this period, the strength of CM4 concrete was modified with an increased amount of fly ash and microsilica, reaching almost the same strength as the CM1 base concrete, and the difference in strength is only 2%. A similar phenomenon was observed in CM2 and CM3 concretes, but in this case, the difference was 8% and 6%, respectively. The test results indicate that the highest flexural strength and tensile splitting strength was obtained in CM1 concrete in which the amount of pozzolans in the form of microsilica and fly ash is 30% of the cement mass. In the microstructural analysis, this concrete was characterized by the most compact C-S-H structure. The presence of fly ash determines the changes in the flexural strength and tensile splitting strength of concrete and limits the action of microsilica in this respect.

Fly ash reduces the pozzolanic reactivity of microsilica in the initial phase of concrete maturation, which affects the reduction in its strength. During the long maturation period, a pozzolanic reaction with the participation of fly ash takes place, which causes an increase in the strength of concrete. Therefore, maintaining the appropriate proportion between cement, microsilica and fly ash allows for obtaining high strength properties both in the early and long maturation period of concrete. This is extremely important due to the effective use of these active additives and ensuring the highest possible strength parameters and durability of concrete. Partial replacement of microsilica with fly ash results in the same compressive strength of CM2 concrete after 56 days as CM4 concrete containing an increased amount of microsilica and fly ash achieves after only 90 days. Based on the obtained test results, it can be concluded that concretes containing fly ash and microsilica in the total amount of 23–33% of the cement mass are characterized by a significant increase in compressive strength after 56 days of maturation, by 20% compared to the strength obtained after 28 days. In turn, concretes containing fly ash and microsilica in the total amount above 40% of the cement mass are characterized by a noticeable increase in compressive strength by 6% after 90 days of maturation compared to the strength obtained after 56 days.

Microsilica seals the structure of the concrete, but fly ash effectively increases its stiffness. This phenomenon is due, among other things, to the activation of pozzolanic processes related to the sealing of cracks occurring in the structure at the interface between the paste and the aggregate. Increasing the microsilica content with reduced fly ash content in CM3 concrete causes a decrease in the mean value of both the initial and stabilized secant modulus of elasticity. This effect is compensated by the combination of increased microsilica and fly ash content in CM4 concrete. Resistance to the penetration of water under pressure into concrete is directly related to the microsilica content and increases with increasing its addition in concrete. Increasing the microsilica content by 1.5% of the cement mass increases the tightness of concrete, which consists in reducing the depth of penetration of water under pressure by 5% and water absorption by as much as 59%. These results confirm the superiority of microsilica sealing properties over fly ash, which is due to its pore-filling properties as well as improving the contact zone around cement particles.

The combination of an increased amount of microsilica to 14.7% and fly ash to 24.7% of the cement mass increases the flexural strength of CM4 concrete and its tightness more effectively than in the case of using an increased amount of microsilica to 14.7% while reducing the amount of fly ash to 8.3% in CM3 concrete. This is the effect of reducing the pozzolanic activity of microsilica due to the presence of fly ash in the initial phase of concrete maturation, which results in a decrease in the flexural strength of concrete with a simultaneous progression of the pozzolanic reaction with the participation of fly ash components, which then proceeds slowly and causes an increase in strength over a long period of maturation.

It should be noted that during the long maturation period, the use of an increased amount of fly ash as a partial substitute for microsilica does not cause deterioration of the mechanical properties of concrete and leads to a significant reduction in the cost of making the concrete mix. The combination of the increased addition of microsilica in the amount of 14.7% of the cement mass and fly ash in the amount of 24.7% increases the delay in achieving both compressive and flexural strength up to 90 days of maturation. Increased microsilica content with a significant reduction in fly ash may lead to a decrease in the compressive strength of concrete during a long maturation period, which is observed in the case of CM3 concrete after 90 days. It should be noted, however, that the strength of CM3 concrete after 90 days of maturation is still 12% higher than the strength of this concrete determined after 28 days of maturation.

Microsilica and fly ash can be ecological and effective additives to ultra-high-performance concretes in terms of physical and strength properties assuming a constant amount of cement, water and superplasticizer for UHPC concrete mixtures modified with the addition of microsilica and fly ash, and allowing for the observation of their direct effect on the physical properties of the concrete mixture and the mechanical properties of hardened concrete, as well as on the cost of the concrete mixture components. Based on the results obtained, the following conclusions can be drawn:

1. The best strength parameters during normal and long periods of maturation, both in terms of compression and flexural and tensile splitting, were obtained for CM1 concrete in which the addition of microsilica and fly ash constitutes 13.3% and 16.7% of the cement mass, respectively.
2. CM3 concrete, containing the largest addition of microsilica, is characterized by the highest compressive strength in a short maturation time and the best resistance to the penetration of water under pressure.
3. The lowest cost while maintaining high strength parameters over a long maturing period, i.e., over 90 days, is characterized by concrete CM2 containing the largest amount of fly ash.
4. The use of increased amounts of fly ash and microsilica in CM4 concrete provides the best protection for concrete against the absorption of water under pressure, but it is also the most expensive.

## Figures and Tables

**Table 1:** Components of concrete mixes, w/c and w/b ratio.

Component	CM1		CM2		CM3		CM4	
	kg/m^3^	% c	kg/m^3^	% c	kg/m^3^	% c	kg/m^3^	% c
Cement (C)	550	100.0	550	100.0	550	100.0	550	100.0
Fly ash (FA)	91.6	16.7	146.6	26.7	45.8	8.3	146.6	26.7
Microsilica (MS)	73.3	13.3	36.7	6.7	81.0	14.7	81.0	14.7
Basalt (B1) 0.5–1.0 mm	429.6	78.1	451.3	82.1	438.6	79.7	399.5	72.6
Basalt (B2) 1.0–2.0 mm	633.7	115.2	665.8	121.1	647.1	117.7	589.4	107.2
Sand (S)	435	79.1	455.2	82.8	443.4	80.6	404.6	73.6
Water (W)	138.6	25.2	138.6	25.2	138.6	25.2	138.6	25.2
Superplasticizer admixture (SP)	9.9	1.8	9.9	1.8	9.9	1.8	9.9	1.8
w/c					0.27			
w/b	0.21		0.20		0.22		0.20	

**Table 2:** Results of concrete mix consistency testing.

Concrete Mix Series	Cone Slump (mm)	Class of Consistency
CM1	210	S4
CM2	90	S2
CM3	175	S4
CM4	210	S4

**Table 3:** Cost indicators for component materials of concrete mixes.

**Component**	$\upsilon_{k}$ **Equation (8)**	**CM1**		**CM2**		**CM3**		**CM4**	
		$\mu_{CM1,k}$ **Equation (9)**	$V_{CM1,k}$ **Equation (10)**	$\mu_{CM2,k}$ **Equation (9)**	$V_{CM2,k}$ **Equation (10)**	$\mu_{CM3,k}$ **Equation (9)**	$V_{CM3,k}$ **Equation (10)**	$\mu_{CM4,k}$ **Equation (9)**	$V_{CM4,k}$ **Equation (10)**
Cement (CEM)	1 ^1^	1.000	1.000	1.000	1.000	1.000	1.000	1.000	1.000
Fly ash (FA)	0.25	0.167	0.042	0.267	0.067	0.083	0.021	0.267	0.067
Microsilica (MS)	5.0	0.133	0.666	0.067	0.334	0.147	0.736	0.147	0.736
Basalt (B1) 0.5–1.0 mm	0.1	0.781	0.078	0.821	0.082	0.797	0.080	0.726	0.073
Basalt (B2) 1.0–2.0 mm	0.1	1.152	0.115	1.211	0.121	1.177	0.118	1.072	0.107
Sand (S)	0.05	0.791	0.040	0.828	0.041	0.806	0.040	0.736	0.037
Superplasticizer admixture (SP)	8.0	0.018	0.144	0.018	0.144	0.018	0.144	0.018	0.144
$W_{CMj}$ Equation (11)			2.085		1.789		2.139		2.164
$\psi_{CMj}$ Equation (12)		1.000		0.858		1.026		1.038	

^1^ The assumed price of cement is USD 180 per ton.

## Data Availability

The original contributions presented in this study are included in the article. Further inquiries can be directed to the corresponding author.
